# Analysis of complete mitochondrial genome sequences increases phylogenetic resolution of bears (Ursidae), a mammalian family that experienced rapid speciation

**DOI:** 10.1186/1471-2148-7-198

**Published:** 2007-10-24

**Authors:** Li Yu, Yi-Wei Li, Oliver A Ryder, Ya-Ping Zhang

**Affiliations:** 1 Laboratory for Conservation and Utilization of Bio-resource, Yunnan University, Kunming 650091, China; 2 State Key Laboratory of Genetic Resource and Evolution, Kunming Institute of Zoology, Kunming 650223, China; 3 Conservation and Research for Endangered Species, Zoological Society of San Diego, PO Box 551, San Diego, CA 92112, USA

## Abstract

**Background:**

Despite the small number of ursid species, bear phylogeny has long been a focus of study due to their conservation value, as all bear genera have been classified as endangered at either the species or subspecies level. The Ursidae family represents a typical example of rapid evolutionary radiation. Previous analyses with a single mitochondrial (mt) gene or a small number of mt genes either provide weak support or a large unresolved polytomy for ursids. We revisit the contentious relationships within Ursidae by analyzing complete mt genome sequences and evaluating the performance of both entire mt genomes and constituent mtDNA genes in recovering a phylogeny of extremely recent speciation events.

**Results:**

This mitochondrial genome-based phylogeny provides strong evidence that the spectacled bear diverged first, while within the genus *Ursus*, the sloth bear is the sister taxon of all the other five ursines. The latter group is divided into the brown bear/polar bear and the two black bears/sun bear assemblages. These findings resolve the previous conflicts between trees using partial mt genes. The ability of different categories of mt protein coding genes to recover the correct phylogeny is concordant with previous analyses for taxa with deep divergence times. This study provides a robust Ursidae phylogenetic framework for future validation by additional independent evidence, and also has significant implications for assisting in the resolution of other similarly difficult phylogenetic investigations.

**Conclusion:**

Identification of base composition bias and utilization of the combined data of whole mitochondrial genome sequences has allowed recovery of a strongly supported phylogeny that is upheld when using multiple alternative outgroups for the Ursidae, a mammalian family that underwent a rapid radiation since the mid- to late Pliocene. It remains to be seen if the reliability of mt genome analysis will hold up in studies of other difficult phylogenetic issues. Although the whole mitochondrial DNA sequence based phylogeny is robust, it remains in conflict with phylogenetic relationships suggested by analysis of limited nuclear-encoded data, a situation that will require gathering more nuclear DNA sequence information.

## Background

**Figure 1:**
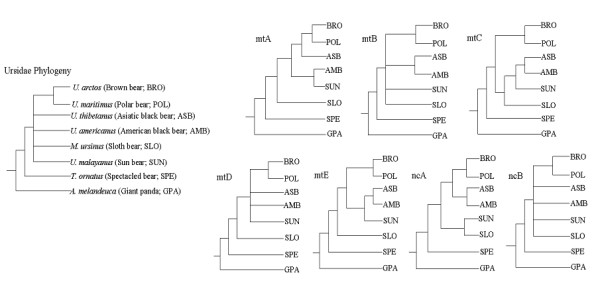
Long-standing unresolved Ursidae phylogeny (tree in the left) and competing hypotheses proposed based on previous sequence data (mtA-E and nuA-B). Trees were constructed from (mtA) combined analysis of partial control region, 12SrRNA, CYTB, tRNA^Pro^, and tRNA^Thr ^mt genes [11], (mtB) MP analysis of complete CYTB, tRNA^Pro^, and tRNA^Thr ^mt genes [12], (mtC) NJ analysis of complete CYTB, tRNA^Pro^, and tRNA^Thr ^mt genes [12], (mtD) combined analysis of partial control region, CYTB, ND4, ND5, COII, and 16SrRNA mt genes [8], (mtE) combined analysis of partial control region, 12SrRNA, complete CYTB, tRNA^Pro^, and tRNA^Thr ^mt genes [13], (nuA) combined analysis of interphotoreceptor retinoid binding protein (IRBP) exon1 and transthyretin (TTR) intron 1 nuclear genes [13], and (nuB) combined analysis of four type I sequence-tagged sites (STS) and IRBP exon 1 nuclear genes [60].

The Ursidae is a major family of the order Carnivora, comprising eight species. They are generally classified into three genera: *Ailuropoda *(giant panda), *Tremarctos *(spectacled bear), and *Ursus *(brown, polar, sloth, sun, and Asiatic and American black bears) [1-3]. Despite the small number of ursid species, bear phylogeny has long been a focus of study due to their conservation value, as all bear genera have been classified as endangered at either the species or subspecies level. The ultimate recognition of the giant panda *Ailuropoda melanoleuca *as the most basal offshoot in the bear family, after more than a century of debate since this species' discovery in 1864, was one of the most spectacular events in the history of modern phylogenetics [4-8]. This achievement also sparked controversy regarding the relationships among the other members of Ursidae and the possibility of an extremely recent radiation for genus *Ursus *[6,8,9] (6 million years ago). Up to now, only the subsequent divergence of *Tremarctos ornatus *(the spectacled bear) to the giant panda, and the sister grouping of *Ursus arctos *(the brown bear) and *Ursus maritimus *(the polar bear) have been unambiguously accepted. All other relationships within the genus *Ursus *remained an unresolved polytomy (Figure 1).

Finding a valid genetic marker that offers sufficient variation to distinguish among recently divergent species posed a major challenge to advancing the understanding of ursid phylogeny. Previous investigations of phylogenetic relationships among the bear species mainly utilized analysis of portions of a single mitochondrial (mt) gene or a small number of mt genes [8,10-13]. In general, mtDNA accumulates mutations at a relatively faster rate and has a shorter expected coalescence time than other types of sequence data, e.g. nuclear DNA, thus making it particularly useful for revealing closely spaced branching events [14-17]. However, none of the previous mt analyses provided conclusive resolution for this low-level phylogeny. Additionally, analyses of different genes within the mt genome have resulted in inconsistent branching patterns being reported in the Ursidae (Figure 1A–E). Recently, several lines of evidence have demonstrated that using sufficiently large amounts of mtDNA sequence data, e.g. the whole mt genome, is a powerful way to ameliorate the discordances and poor resolution that plague analyses based on single genes or segments [16,18-22]. Thus, as a further step toward the understanding of Ursidae phylogeny, it was highly desirable to address this evolutionary question from a mitogenomic perspective.

On the basis of the most comprehensive molecular data set assembled to date for the Ursidae, the present work revisits the contentious relationships within genus *Ursus *by analyzing complete mt genome sequences from all representatives of bears and, for the first time, evaluates the performance of both entire mt genomes and constituent mtDNA genes in recovering a phylogenetic tree within a rapid, recent radiation. The improved reconstruction of ursid relationships utilizing the entire mt genome also permitted a refined dating of evolutionary divergence among these bear species. Our research thus not only generates a strong Ursidae phylogenetic framework for future validation using additional evidence but, most significantly, provides a model system against which to examine the usefulness of mt genomes for resolving difficult phylogenies with rapid species radiation.

## Results

### Sequence Characteristics

The general characteristics of eight bear mt genomes are summarized in Table 1. These complete mt genomes range from 16,746–17,020 bp in size. Length differences are largely due to the variation in copy number of tandem repeated sequences in the conserved sequence block (CSB) domains of the mt control region. All genomes share not only 13 protein-coding genes, 22 tRNAs genes, 2 rRNAs, and a control region, but also the same gene order. The overall average nucleotide composition of bear mt genomes is A = 30.9%, C = 25.0%, G = 15.6%, and T = 28.5%. The constancy of nucleotide base composition was examined using the chi-square test with PUZZLE for different subsets of mt genome (defined in Methods), and heterogeneous nucleotide composition (p < 0.05) was observed in the combined protein-coding gene dataset and the complete dataset (as described below). The K2P distances [23] among seven ingroup taxa calculated using MEGA3 [24] range from 2.3 to 19.9% for the protein-coding dataset (average 12.8%), from 1.2 to 10.1% for the rRNA dataset (average 6.1%), from 1.3 to 8.7% for the tRNA dataset (average 5.2%), from 5.0 to 18.9% for the control region (average 10%), and from 2.2 to 17.0% for the complete dataset (average 10.7%).

**Table 1:** General characteristics of eight bear mt genomes

**Taxa**				**Genome Length(bp)**					**G+C nucleotide content (%)**				
				
Genus	Scientific Name	**Sample Source**	**Accesion No.**	total	protein coding	rRNAs	tRNAs	control region	total	protein-coding	rRNAs	tRNAs	control region
*Ursus*	*U. arctos*	----	AF303110 [38]	17,020	10,882	2,541	1,511	1,578	40.8	41.5	40.5	35.9	41.6
	*U. maritimus*	----	AF303111 [38]	17,017	10,882	2,542	1,511	1,575	40.8	41.4	40.6	36.2	42.0
	*U. thibetanus*	Yunnan Province, China	EF196661 (this study)	16,795	10,877	2,548	1,512	1,346	40.7	41.4	40.3	35.9	40.6
	*U. americanus*	----	AF303109 [38]	16,841	10,882	2,545	1,512	1,396	40.4	41.0	40.2	36.4	40.3
	*U. malayanus*	Yunnan Province, China	EF196664(this study)	16,783	10,882	2,546	1,512	1,337	40.7	41.4	40.2	35.9	40.2
	*U. ursinus*	San Diego Zoo, USA	EF196662 (this study)	16,817	10,882	2,545	1,511	1,371	41.6	42.3	40.9	36.9	41.7
*Tremarctos*	*T. ornatus*	San Diego Zoo, USA	EF196665 (this study)	16,766	10,882	2,553	1,510	1,315	41.1	41.9	40.1	35.7	43.0
*Ailuropoda*	*A. melandeuca*	Sichuan Province, China	EF196663 (this study)	16,746	10,880	2,551	1,515	1,286	38.5	38.3	39.0	37.0	41.1

### Reconstructing the Phylogenetic Relationships

**Figure 2:**
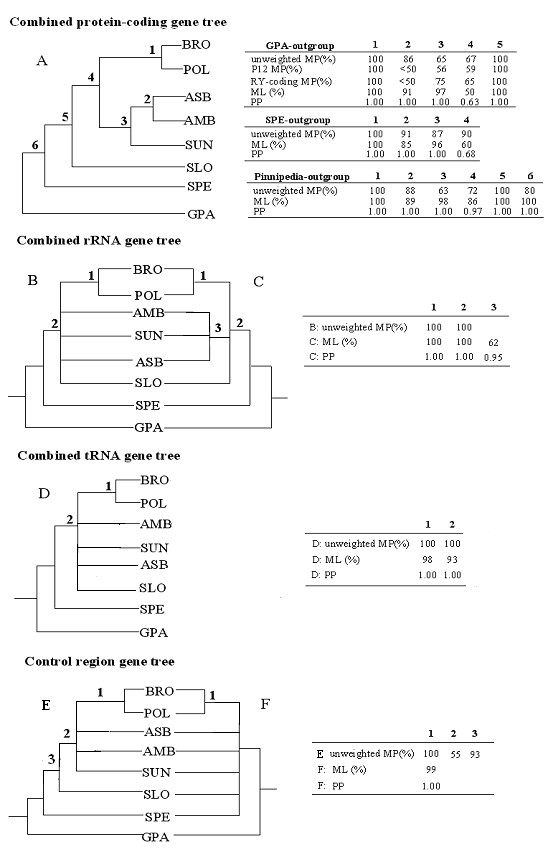
Phylogenetic trees and nodal supports (groups that received more than 50% BS or 0.6 PP were retained) based on analyses of different subsets of mt genome.

The combined data set of 12 protein-coding genes (10882 aligned nucleotide sites; 3534 variable and 1970 parsimony-informative) produced a single most-parsimonious tree of 5512 steps without MP weighting (Figure 2A). In this tree, the spectacled bear diverged earliest (MP BS = 100%), followed by the sloth bear (MP BS = 67%). Sister-group relationships were indicated between the Asiatic black and the American black bear (MP BS = 86%), as well as between the brown and the polar bear (MP BS = 100%), while the sun bear clustered with the two black bears (MP BS = 65%). Because the giant panda sequence deviates significantly from the mean nucleotide frequency on the 3rd codon position by a 5% chi-square test (p < 0.05), the phylogenetic analysis was therefore also conducted under different weighting schemes, including P12 and RY-coding methods (see Methods). In all cases, an identical topology to that of unweighted analysis was obtained, but the close relatedness between the Asian black and American black bears was less well supported (MP BS < 50%; Figure 2A). Generally, better resolution and stronger bootstrap supports were obtained from DNA datasets that included all substitutions than from those subjected to the weighting. As an alternative attempt to evaluate the effect of compositional bias on the reconstructed tree, we reanalyzed the data without the giant panda and used spectacled bear for rooting. This approach provided a data set without significant base composition variation (p > 0.05). Interestingly, the resulting tree topology remained constant but there was a noticeable effect on the nodal support, where all relationships in the tree were robustly identified (MP BS > 85%; Figure 2A). Particularly, BS for the positions of the sloth bear and the sun bear increased to 90% and 87%, respectively. ML and partitioned Bayesian analyses (using distinct models and rates for each protein gene) of the dataset, whether the giant panda or the spectacled bear was used as outgroup, showed the same tree topology as Figure 2A. All nodes in the ML and Bayesian trees received high BP (≥ 85%) and PP (≥ 0.99) except for the position of the sloth bear. Tables in Figure 2A illustrate the confidence level of the nodal relationships under all analytical approaches.

Both of the combined RNA data sets (rRNAs and tRNAs) demonstrated reduced resolving power for phylogenetic inference compared to protein-coding gene analysis (Figure 2B–D). The aligned rRNA sequences (combined 12S and 16SrRNA genes) were 2574 bp in length, of which 480 nucleotide sites were variable and 222 were parsimony-informative. MP analysis yielded two equally most-parsimonious trees of 683 steps. One of them is topologically identical to the protein-coding gene tree shown in Figure 2A. Although most relationships collapsed on the 50% majority-rule consensus of the two parsimonious trees (Figure 2B), the two black bears and the sun bear formed a clade on the ML (ML BS = 62%) and partitioned Bayesian trees (distinct models and rates for two rRNA genes; PP = 0.95; Figure 2C), a relationship also supported in the protein-coding gene analysis. Interestingly, when stem-loop secondary structures were considered, the single most-parsimonious tree (495 steps) based on the loop region (1283 bp) identified the same topology as Figure 2A. In contrast, 50% majority-rule consensus of three equally most-parsimonious trees (186 steps) based on the stem region (1291 bp) recovered a tree topology that differed in placing the Asiatic black bear as basal to the reset of *Ursus*, while joining the American black bear, the sun bear, and the sloth bear on a common branch (data not shown). However, nodal supports for most relationships in the stem and loop trees are below 50%, with the exception of the earliest branching of the spectacled bear among the in-group and the close association of the brown and polar bears (MP BS = 100%).

The tRNA data set (combined 22 tRNA genes) contained 1518 bp of aligned sites, of which 256 were variable and 100 were parsimony-informative. Parsimony analysis produced a most-parsimonious tree of 341 steps with a different topology from those of protein-coding and rRNA gene analyses. According to this tree, the sloth bear was grouped with the Asiatic black bear, and they are placed as a clade sister to the lineage leading to the American black bear and the sun bear. However, in this tree only the basal position of the spectacled bear, and the close association of the brown bear and the polar bear, were convincingly supported (MP BS = 100%; Figure 2D); none of the other relationships received MP BS larger than 50%. ML and Bayesian analyses produced a similar tree topology and nodal support as the MP tree.

In the control region of the mt genome, tandem repeated sequences and ambiguously aligned regions were excluded, leaving 1008 positions in the phylogenetic analysis. 316 were variable and 124 were parsimony-informative. MP reconstruction yielded a most-parsimonious tree of 463 steps. The striking topological difference from protein-coding and RNA gene analyses was the positioning of the sun bear, Asian black bear, and American bears as the successive sister taxa to the clade consisting of the brown and polar bears. However, only the branch that separates the spectacled bear from *Ursus *and the basal diverging of the sloth bear within *Ursus*, and the affinity of the brown and polar bears, received >50% bootstrap support (Figure 2E). ML and Bayesian analyses of the same data set found a large unresolved polytomy leading to most bear species, and only the sister-grouping of the brown and polar bears was strongly supported (ML BS = 99% and PP = 1.00; Figure 2F).

**Figure 3:**
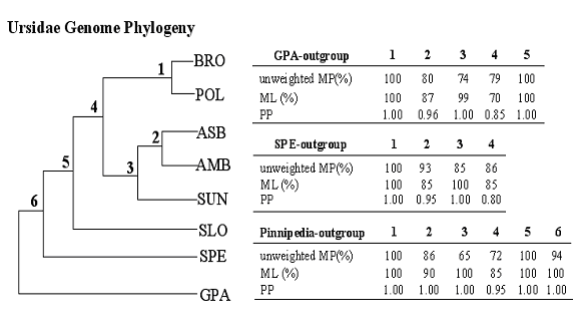
Mt genome tree and nodal supports.

To maximize the amount of phylogenetic information, we also pooled all mt protein-coding genes, RNA genes, and control regions to form a single data set with a length of 15982 aligned sites for "genome phylogeny" reconstruction, of which 4586 were variable and 2416 were parsimony-informative. A unique topology exactly identical to that produced by the combined protein-coding gene analysis, but with higher statistical nodal supports, was obtained by all three analytical approaches (Figure 3; 70–100% BS and 0.85–1.00 PP). The tree topology was not affected by exclusion of the giant panda sequence (p < 0.05 with PUZZLE) and moreover, by rooting the tree with the spectacled bear, the supports for most branches increased to convincing statistical significance (≥ 85 BS and ≥ 0.95 PP), increasing our confidence in the result. The complete mtDNA genome-based phylogeny of the Ursidae incorporates the largest amount of phylogenetically informative sequence-based characters and provides the most robust tree for this carnivore family in terms of resolved topology and support for nodes.

### Assessing the Performance of Individual Genes

**Figure 4:**
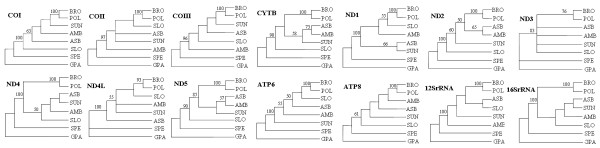
Phylogenetic trees based on individual mt genes. Only >50% MP BS are indicated on the branches.

The determination of complete mt sequences from all bear species affords the opportunity not only to utilize these data to estimate the phylogeny of the Ursidae but also, to examine characteristics of the evolution of mitochondrial protein coding and non-coding genes. It is of interest to evaluate their individual performance in supporting the complete mtDNA-based phylogeny within such a recent radiation involving specializations to a variety of habitats and reproductive patterns. For this reason, unweighted MP analysis was also performed on protein-coding and rRNA genes individually (Figure 4; only >50% MP BS are indicated on the branches). The earliest split of the spectacled bear and the grouping of the brown bear and polar bear, which are the only two strongly supported hypotheses in Ursidae phylogeny to date, are observed in all of these single-gene trees (61–100% and 76–100% MP BS, respectively) whereas the position of the other bear species in *Ursus *was either not recovered at all or varied considerably with little or no nodal support. Among these single gene trees, only the CYTB and ND5 trees have the same branching order as that from the combined all gene analysis (Figure 3).

**Figure 5:**
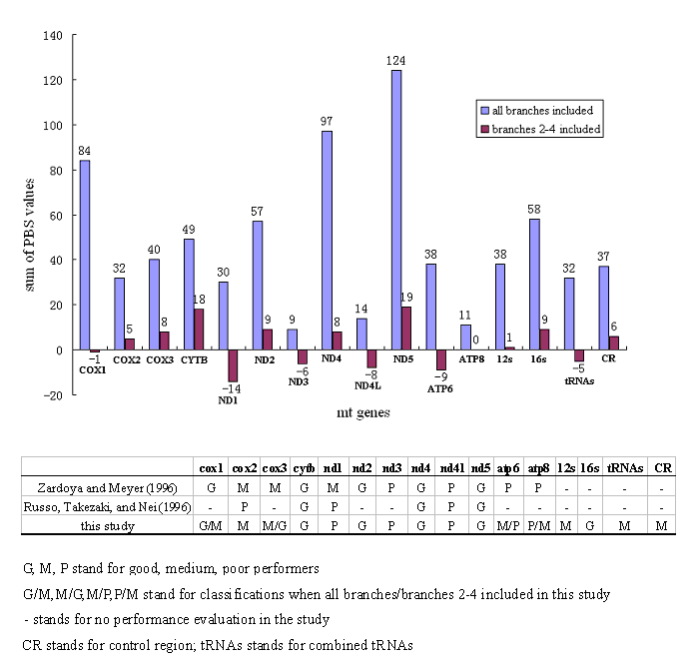
Results of partitioned Bremer support (PBS) analyses to each node on the mt genome tree and comparisons of phylogenetic performance of mt genes among the studies.

To better assess which grouping in whole-genome phylogeny was supported by different parts of the mt genome (i.e., protein-coding, RNA genes, and control region), we performed partitioned Bremer support (PBS) analysis and the result is shown in Figure 5. The partial Bremer index for each data partition is determined by subtracting the number of steps for that partition in the most parsimonious tree from the number of steps for that partition in the shortest tree lacking the node in question [25]. Partial decay indices may be either positive or negative for an individual data partition. PBS analysis indicated that among the 16 gene partitions examined (Figure 5), the ND5 gene provides the greatest contribution to the complete gene tree resolution (124/750 = 16.53%) while the ND3 gene contributes the least (9/750 = 1.30%). From the results of PBS analysis, we find that all genes allocated Bremer supports predominately in the traditionally well-established relationships, i.e., branches 1 and 5 in the tree (94.93% in total; data not shown). Leaving these two branches out of the analysis, the ND5 gene still provided the largest proportion of PBS values (19/40 = 47.50%) compared to those of the remaining branches (Figure 5). In contrast, the ND1 gene provided the highest conflict values (-14/40 = -35.00%). Whether branches 1 and 5 were included or not, PBS analyses give the rough appearance of relatively superior performance of ND5, ND4, CYTB, 16SrRNA, and ND2 genes, and on the other hand, of the poor utility of ND3, ND4L and ND1 genes. Combined tRNAs, COX2, 12SrRNA genes, and control region are intermediate (Figure 5).

### Dating the Evolutionary Divergences

Results of a likelihood ratio test of the molecular-clock hypothesis for the five data sets, as defined for the phylogenetic analysis, are presented in Table 2. The combined protein-coding data set was used in estimating divergence times of Ursidae radiation, in view of its relatively good tree resolution and constant evolutionary rates across all taxa. By applying a minimum paleonotological date of separation between the giant panda and the other bear species of 12 million years ago [26,27] (MYA), which has also been used as an established reference in all previous studies of Ursidae [8,12,13], we inferred that the divergence of the spectacled bear from the ursine clade occurred at 10.91 MYA (95% confidence intervals = 9.93–11.89 MYA). The six closely related bears in genus *Ursus *began their recent radiation from a common ancestor at 6.34 MYA (95% confidence intervals = 5.95–6.73 MYA; node 5 in Figure 3). Similar thinking applied to the genus *Ursus *suggests a divergence time between the brown and polar bears and the two black bears/sun bear of 6.13 MYA (95% confidence intervals = 5.54–6.72 MYA; node 4), and that between the sun bear and the two black bears of 5.673 MYA (95% confidence intervals = 5.09–6.26 MYA; node 3). The dating for the divergence between the Asian black and American black bear was 5.19 MYA (95% confidence intervals = 4.6–5.78 MYA; node 2), and that between the brown bear and polar bear 1.32 MYA (95% confidence intervals = 0.93–1.71 MYA; node 1). Table 3 shows these divergence time evaluations and also those in previous studies for comparison.

**Table 2:** Results of likelihood ratio test of the molecular-clock hypothesis

	combined protein-coding gene	combined rRNAs gene	combined tRNAs gene	control region	combined all gene
Log L without clock	-36253.65	-6614.67	-3682.41	-3351.94	-50256.75
Log L with clock	-36258.55	-6616.48	-3685.10	-3431.73	-30265.12
significance level (p)	0.13	0.73	0.49	<0.01	<0.05

**Table 3:** Divergence time evaluation of Ursidae family (Mya)

	fossil records^a^	protein electrophoresis analysis from Goldman, Giri and O'Brien (1989) [6]	partial mt gene analysis from Talbot and Shields (1996) [12]	two nuclear gene analysis from Yu et al (2004) [13]	mt genome analysis from this study
*T. ornatus *separation	5–7	10–15	12–13	6–8	10.91
six *Ursine *bears (or *U. ursinus*) divergence (node 5)	4–6	4–8	5–7	2–5	6.34
five *Ursine *bears (except *U. ursinus*) divergence (node 4)	-	-	6	-	6.13
*U. malayanus *divergence (node 3)	0.2–1 or 5–10	-	5	-	5.67
*U. thibetanus *– *U. americanus *split (node 2)	1–3.5	-	5	-	5.19
*U. arctos *– *U. maritimus *split (node 1)	0.07–0.1	2–3	1–2	1–1.5	1.32

- no divergence time evaluation in the study

a from Kurten 1968 [9]; Thenius 1979 [27]; Kurten and Anderson 1980 [35]; Savage and Russell 1983 [37]; Wayne et al. 1991 [26]

## Discussion

Among mammalian phylogenies, those characterized by rapid species radiations have long been one of the most plaguing and challenging problems in species tree reconstruction [28]. This is the first study utilizing data from whole mitochondrial genome sequences from ursids, an approach that allows increased phylogenetic resolution of the Ursidae family, whose origin can be traced back to the extremely recent mid-Miocene[6,9] (15–20 MYA). Previous molecular studies relevant to Ursidae phylogeny provided either an inconsistent view on the issue or weak statistical support for discriminating alternative hypotheses (Figure 1). The branching event following the divergence of the spectacled bear has long been a large unresolved polytomy leading to six *Ursus *species, of which only the sister-relationship between the brown bear and polar bear was unanimously favored. The close relatedness of brown bear and polar bear, as well as the paraphyletic association between mtDNA of these two bears has been upheld in previous studies [29-31]. More sequences of brown bear and polar bear included in the future research will help test further the earlier observations. In sum, the long-standing lack of full resolution within Ursidae may be primarily due to the low level of variation harbored in much shorter sequences than those used in this study.

Based on the largest available mt data set from Ursidae, our genome phylogeny provides strong evidence that within genus *Ursus*, the sloth bear is the sister taxa of all the other five ursines, and that the latter group is divided into the brown bear/polar bear and the two black bears/sun bear assemblages, upholding and strengthening the hypothesis drawn by our previous analysis of the five fragments of mtDNA [13] (Figure 1E). Alternative hypotheses for a mitochondrial sequence based phylogeny are not supported when the entire mitochondrial DNA sequence information is utilized. In particular, when nucleotide base compositional bias introduced by the giant panda outgroup was removed from our analysis and the spectacled bear was used for rooting, the overall Ursidae relationships recovered have the largest statistical support in comparison to all other previously proposed hypotheses. To further examine the sensitivity of our tree topology to outgroup choice, we selected Pinnipedia. This superfamily in Carnivora includes the Otariidae, Phocidae, and Odobenidae families, members of which have been used as alternative roots in earlier studies of bears [8,12].

Combined protein-coding genes (10888 aligned sites, 4522 variable and 3245 parsimony-informative) and all mt genes (15017 aligned sites, 5642 variable and 3926 parsimony-informative; control region was not included due to alignment difficulty) analyses of the eight bear species using available Pinnipedia mt genomes as outgroups (Accession No. AJ428576, AJ428578 and X63726; [32,33]) gave an identical tree topology to that obtained using the giant panda as the outgroup. Support levels for most branches were similar to those estimated with the giant panda outgroup except for an increased ML BS (≥ 85) and PP (≥ 0.95 PP) of placement of the sloth bear (Figure 2A and 3). A chi-square test of composition stationary showed that both Pinnipedia and the giant panda have deviant base composition with respect to the other bears (p < 0.05), a circumstance that might have a negative impact on the branch supports in MP analysis. Thus, our genome phylogeny was robust with either outgroup, though the spectacled bear, exhibiting the least phylogenetic noise, appears a more favorable outgroup for Ursidae in terms of overall support levels. Taken together, the present genome result significantly resolved the conflict between those trees using partial mt genes [8,11-13] and represents the most probable explanation of bear evolution.

Nevertheless, a more in-depth understanding of the Ursidae relationships will definitely benefit from the addition of independent sequence data, considering that the genome phylogeny obtained here is based on a single and haploid linkage unit. The necessity of including other unlinked genes for phylogenetic resolution of the Ursidae is also illustrated by the fact that our recent study on two nuclear genes, transthyretin (TTR) and interphotoreceptor retinoid binding protein (IRBP), has united the sloth bear and the sun bear as sister taxa with high statistical support [13] (Figure 1 nuA), a relationship not consistent with mt gene analyses, including the present study. In mt trees, the sloth bear was mostly placed as the earliest diverging taxa among the six ursine species [8,11,13, and the present result], and the sun bear closer to the American black bear [11], the brown bear/polar bear clade [MP phylogeny in 12; ML phylogeny in 8], or the clade including the two black bears (NJ phylogeny in 12; 13 and the present result). Notably, the sister relationship between the Asian and American black bears, previously proposed on paleontological and morphological grounds [34,35] was reinforced by consistent recovery from both our mt genome and nuclear analysis. Such a grouping has also been retrieved previously with moderate support by mt analysis of complete CYTB and 2 tRNAs [12], as well as the addition of the partial D-loop region [13]. Thus, the placements of the sun bear and the sloth bear represent are the most obvious discrepancy observed in the mt and nuclear trees comparisons. Our genome analysis has established a very useful benchmark that can be tested with future independent evidence.

Our genome analyses provide important insights into not only Ursidae phylogeny, but also the phylogenetic utility of different mt genes. Our data add to the well-studied performance of individual mt genes, mostly protein-coding genes, for estimating phylogeny of deep divergence [16,36], we are interested to see their relative efficiencies, adding mt RNA genes and control region as well, in those of extremely recent split. Our results suggest that combined mt protein-coding genes are more informative than the other subsets of mt genes regarding the lower-level bear relationships resolution. Only by combining all genes is it possible to reach a fully-resolved tree with moderate to strong support from MP, ML, and Bayesian methods of analysis. Ranking single genes by their respective contribution to the total PBS values of the genome tree, as a rough indicator of phylogenetic utility, reveals that some genes, such as ND5, ND4, CYTB, ND2, and 16SrRNA are better indicators of Ursidae evolution than are other genes, such as ND3, ND4L, and ND1 (Figure 5). Our results add to previous findings from Zardoya and Meyer (1996) [16] and Russo, Takezaki, and Nei (1996) [36] that did not included concatenated tRNAs, 2 rRNAs, and control regions in the evaluation of phylogenetic performance, and also agree globally with their conclusions about the rough classification of 12 mt protein-coding genes into good, medium, and poor categories. These conclusions are upheld even though significantly different evolutionary time frames between our studies and theirs (i.e., distantly related vertebrates) (Figure 5) are involved. In this sense, general knowledge of phylogenetic values of the mt genes makes it possible to preselect subsets of mt genes for different-level phylogenetic questions in the case of mt genomes unavailable. In fact, in some previous studies, Ursidae phylogenies based on the combined analysis of a few mt genes have also, to a degree, demonstrated the potential valuable information as those based on complete genome analysis [8,12].

Ursidae has one of the most extensive fossil records of extant Carnivora families [9,26,27,35,37]. Given a good fossil documentation and strongly supported phylogenetic relationships from the largest available data set, it is of interest to draw a comparison between the present mitogenomic dating results and those from previous paleontological and molecular evidence (Table 3). According to estimates based on our genome data, all the separation times within genus *Ursus *appear to have occurred in the Late Miocene or Early Pliocene (5–6 MYA), except that in the Early Pleistocene the most closely related bear species, the brown and polar bears diverged. The rather recent origins and rapid succession of these bear lineages are in line with the observation that most short mtDNA sequences used in previous studies lack sufficiently strong phylogenetic signals and provide limited resolving power for recovering a strongly supported Ursidae phylogeny. As is seen in Table 3, our estimates of the diversification of the Ursidae family was more in agreement with those obtained with partial mt genes analysis [12] and protein electrophoresis analysis [6] than those obtained with the fossil record [9,26,35]and nuclear sequence analysis [13], which are slightly younger than the present results.

## Conclusion

Identification of base composition bias and utilization of the combined data of whole mitochondrial genome sequences has allowed recovery of a strongly supported phylogeny that is upheld when using multiple alternative outgroups for the Ursidae, a mammalian family that underwent a rapid radiation since the mid- to late Pliocene.

The suggestion of Delisle and Strobeck (2002) [38] that application of mitogenomic datasets would be likely to be useful for distinguishing nodes resulting from rapid radiation episodes such as the ursine speciation events is validated by these findings. It remains to be seen if the reliability of mt genome analysis will hold up in studies of other difficult phylogenetic issues. Although the whole mitochondrial DNA sequence based phylogeny is robust, it remains in conflict with phylogenetic relationships suggested by analysis of limited nuclear-encoded data, a situation that will require gathering more nuclear DNA sequence information.

## Methods

### DNA Samples and Sequence Determination

The complete mt sequences of three bear species in genus *Ursus*, the polar bear (*U. maritimus*), the brown bear (*U. arctos*), and the American black bear (*U. americanus*), have been determined in previous studies of genome evolution [38]. Thus, the availability of the other five mt genome sequences from Ursidae was of considerable interest for phylogenetic reconstruction. We extracted total DNA from fresh blood or frozen tissues of the Asiatic black bear (*U. thibetanus*), the sloth bear (*U. ursinus*), the sun bear (*U. malayanus*), the spectacled bear (*Tremarctos ornatus*) and the giant panda (*Ailuropoda melanoleuca*) using standard proteinase K, phenol/chloroform extraction [39].

**Figure 6:**
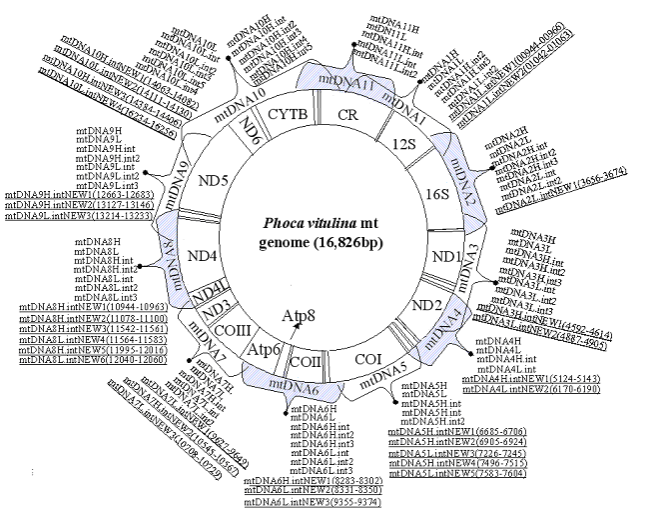
Universal primers [35] and newly designed species-specific primers (underlined) used for amplifying Ursidae mt genomes. Locations of new primers are indicated in brackets and correspond to nucleotide numbers from the harbor seal sequence (Accession No. X63726). Eleven mtDNA fragments (mtDNA1-11) covering the entire mt genome are labeled as described in Delisle and Strobeck (2002) [38].

Mt genome sequences were initially amplified with sets of universal primers (73 in total) described in Delisle and Strobeck's original study (2002) [38]. In the case of poor PCR performance with universal primers, 31 additional species-specific oligonucleotide primers were designed (underlined in Figure 6). Primer sequence information was available upon request. A "touch-down" PCR amplification was carried out using the following parameters: 95°C hot start (5 min), 10 cycles of 94°C denaturation (1 min), 60–50°C annealing (1 min; °C/cycle), 72°C extension (1 min), and finally 25 cycles of 94°C denaturation (1 min), 50°C annealing (1 min), 72°C extension (1 min). The amplified DNA fragments were purified and sequenced in both directions with an ABI PRISM™ 3700 DNA sequencer following the manufacturer's protocol. Mt sequences obtained were checked carefully to ensure that they did not include nuclear copies of mtDNA-like pseudogenes. The exact length of the control region in the mt genome cannot be determined due to the presence of long tandem repeated sequences. Newly determined genomes have been deposited in GenBank under Accession No. EF19661–EF19665.

### Sequence Data Analyses

The complete mt genome sequences of all extant bear species were aligned with program CLUSTAL X [40] and verified by eye. Five data sets comprising (1) all protein-coding genes combined except NADH6 gene, (2) 12S and 16S tRNA genes combined, (3) all 22 rRNA genes combined, (4) control region (CR), and (5) all genes combined were analyzed for phylogenetic reconstruction. The NADH6 gene is excluded from the analyses due to its anomalous nucleotide composition which can confound phylogenetic inferences.

Each of these data sets was subjected to unweighted maximum parsimony (MP) and maximum likelihood (ML) analyses using PAUP *4.0b8 [41]. For MP analyses, we adopted an exhaustive search algorithm with TBR branch swapping. For model-based ML analyses, we introduced hierarchical likelihood ratio tests (hLRT) to compare the goodness of fit of 56 nucleotide substitution models using ModelTest version 3.06 [42]. Once an appropriate model was established, a ML tree was constructed based on this model of sequence evolution. The reliability of phylogenetic relationships was evaluated by bootstrap analysis [43] for MP and ML trees (BS; 1000 replicates for MP and 100 replicates for ML). In addition, we performed a partitioned Bayesian inference (pBI) analysis [44] for phylogenetic reconstruction using MrBayes 3.1 [45], allowing a separate general time reversible (GTR) + I + Γ model and set of parameters for each gene partition, with the assumption that the underlying evolutionary process was potentially different across these partitions. Four Metropolis-coupled Markov chain Monte Carlo (MCMC) analyses were run for 2 million generations, sampling trees every 100 generations. Robustness for branches in pBI analysis was assessed by posterior probability (PP). We also conducted partitioned Bremer support analysis (PBS) [46-48] with TreeRot.v2 [25] to measure the contribution of each data partition to the total Bremer support for the nodes of genome-based tree topology.

To avoid potential tree estimation bias introduced by nucleotide composition [49,50] or saturation, two additional weighting strategies were applied in the analysis of combined 12 protein-coding genes: (1) excluding the 3rd codon positions (P12), and (2) recoding the 3rd codon position nucleotides to two-state categories, R (purine) and Y (pyrimidine), (RY-coding). The RY-coding was used here based on the growing observation that it can greatly improve consistency in phylogenetic resolution by reducing bias from differences in nucleotide composition [51-54]. In the combined analysis, portions of the 12S rRNA and 16S rRNA genes were also partitioned into two separate subsets according to their secondary structures: single-strand stems and base-paired loops [55,56].

The giant panda was used as an outgroup for estimating phylogenetic relationships within genus *Ursus*. To examine if the resulting tree topologies were sensitive to outgroup alteration, we also carried out phylogenetic analysis with Pinnipedia, a non-ursine superfamily in Carnivora, for the rooting.

The molecular clock hypothesis was examined using the likelihood-ratio test [57] with PUZZLE [58,59]. When clock-like behavior was not rejected by the test, the divergence times among them were calculated and compared to previous molecular results and fossil records.

## Competing interests

The author(s) declares that there are no competing interests.

## Authors' contributions

LY and YPZ designed the study. YWL carried out the experiment work. LY performed the analyses. LY, OAR and YPZ prepared the manuscript. All authors read and approved the final manuscript.
